# Intercellular communication in peritoneal dialysis

**DOI:** 10.3389/fphys.2024.1331976

**Published:** 2024-02-08

**Authors:** Li Sheng, Yun Shan, Huibo Dai, Manshu Yu, Jinyi Sun, Liyan Huang, Funing Wang, Meixiao Sheng

**Affiliations:** ^1^ Department of Nephrology, Affiliated Hospital of Nanjing University of Chinese Medicine, Nanjing, China; ^2^ First Clinic Medical School, Nanjing University of Chinese Medicine, Nanjing, China

**Keywords:** peritoneal dialysis, peritoneal fibrosis, intercellular communication, inter-organ communication, crosstalk

## Abstract

Long-term peritoneal dialysis (PD) causes structural and functional alterations of the peritoneal membrane. Peritoneal deterioration and fibrosis are multicellular and multimolecular processes. Under stimulation by deleterious factors such as non-biocompatibility of PD solution, various cells in the abdominal cavity show differing characteristics, such as the secretion of different cytokines, varying protein expression levels, and transdifferentiation into other cells. In this review, we discuss the role of various cells in the abdominal cavity and their interactions in the pathogenesis of PD. An in-depth understanding of intercellular communication and inter-organ communication in PD will lead to a better understanding of the pathogenesis of this disease, enabling the development of novel therapeutic targets.

## 1 Introduction

An estimated 3.8 million people worldwide currently rely on some form of dialysis for treatment of end-stage kidney disease. Peritoneal dialysis (PD) accounts for approximately 11% of patients undergoing dialysis overall (Teitelbaum, 2021). PD utilizes the characteristics of the peritoneum as a semi-permeable membrane to achieve the goal of clearing metabolic products and toxic substances in the body, and correcting water and electrolyte imbalances. The peritoneal cavity is a complex system with both cellular and non-cellular components, primarily consisting of peritoneal mesothelial cells, endothelial cells, pericytes, macrophages, and other immune cells. Upon prolonged exposure to PD, this intricate cellular framework becomes disrupted, initiating extensive remodeling processes aimed at maintaining the fundamental function of the peritoneal membrane. Prolonged peritoneal stress often results in inflammation and angiogenesis, frequently observed in peritoneal fibrosis (Masola et al., 2022).

The understanding of interactions between different cell types within the peritoneal cavity is crucial in the study of peritoneal fibrosis. Intercellular communication is mediated via the exchange of chemical, electrical, and mechanical messages through direct cell-cell transformations or the release of paracrine factors such as cytokines and growth factors (Khattar et al., 2022; Fafian-Labora and O’Loghlen, 2020). An intriguing aspect of cell-cell communication is the discovery that nucleic acids, such as microRNAs, are packaged into extracellular vesicles. These vesicles can be transported from the secreting cells to target cells, where they exert signaling effects. In addition, inter-organ crosstalk in the peritoneal cavity also plays a role in peritoneal fibrosis, especially the network between the peritoneal membrane and immune system (Terri et al., 2021) as well as the peritoneal membrane and gut (Isaza-Restrepo et al., 2018).

**FIGURE 1:**
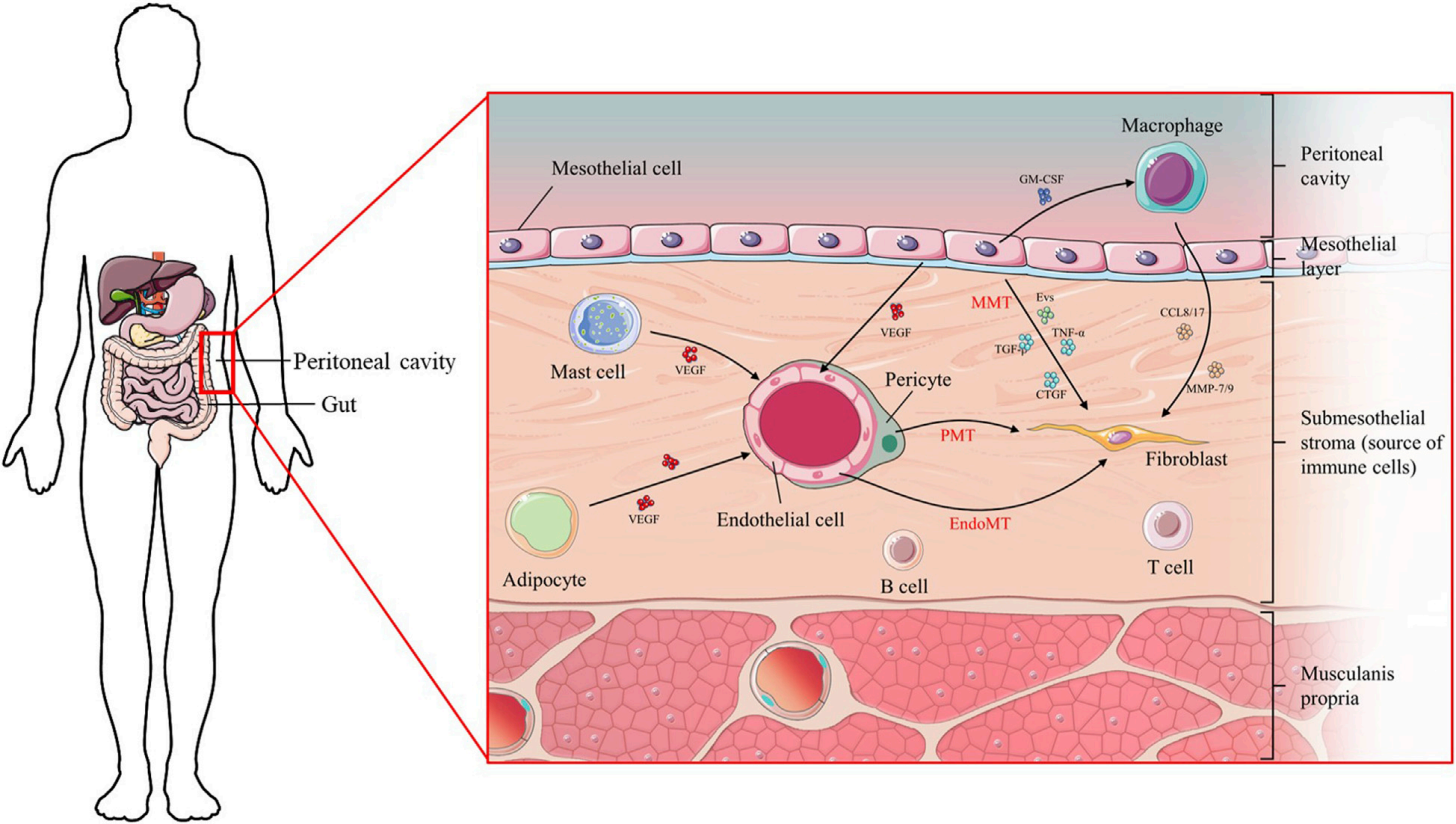
Diagram of the cellular composition of the peritoneal membrane and the mutual location of organs.

This review aims to provide an up-to-date analysis of the latest research on cell-cell communication and inter-organ crosstalk in the context of peritoneal fibrosis (Figure 1). The focus is on recent studies that have shed light on the mechanisms involved in these processes. Understanding the intricate interactions between cells and organs within the peritoneal cavity is essential for comprehending the pathogenesis of peritoneal fibrosis and potentially developing therapeutic strategies to mitigate its progression.

## 2 Intercellular communication

### 2.1 Peritoneal mesothelial cell-(Myo)fibroblast crosstalk

**FIGURE 2:**
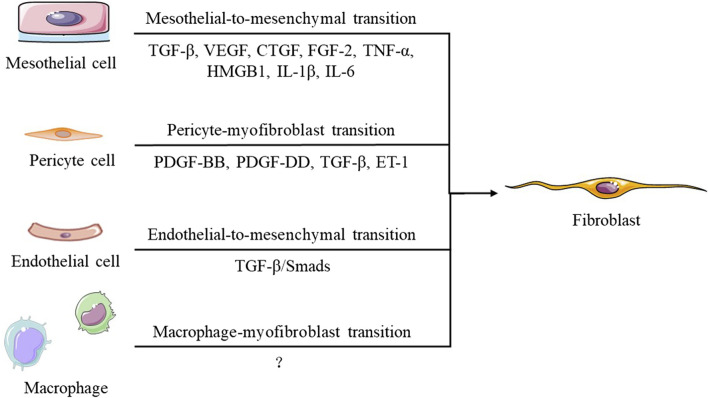
Transition of different cells in the process of peritoneal fibrosis.

Myofibroblasts are the main effector cells involved in peritoneal fibrosis, which alters the normal structure of the peritoneum by inducing submesothelial collagen deposition. There are five main processes through which myofibroblasts are generated (Figure 2): activation and proliferation of resident peritoneal fibroblasts, transformation of bone marrow-derived mesenchymal cells, endothelial cell mesenchymal transformation, pericyte-myofibroblast transition (PMT), and mesothelial-to-mesenchymal transition (MMT) (Witowski et al., 2015). In the process of MMT—previously named epithelial-mesenchymal transition (EMT)—mesothelial cells migrate from the superficial mesothelium to the submesothelial layer, where they produce extracellular matrix (ECM) and promote (myo)fibroblast formation to exacerbate fibrosis. These transformed mesothelial cells lose intercellular junctions, manifested as a decrease in E-cadherin and zona occludens 1 (ZO-1), and instead express (myo)fibroblast proteins such as N-cadherin, vimentin, and α-smooth muscle actin (α-SMA) (Liu F. et al., 2021).

The communication between peritoneal mesothelial cells and myofibroblasts primarily occurs through paracrine factors such as cytokines or growth factors, direct cell-to-cell contacts facilitated by gap junctions, or indirect interactions mediated by ECM proteins. Both cell types have the ability to produce and secrete various chemokines, cytokines, and growth factors such as transforming growth factor *ß* (TGF-β), vascular endothelial growth factor (VEGF) (Kariya et al., 2018), connective tissue growth factor (Sakai et al., 2017), fibroblast growth factor-2 (FGF-2) (Kopytina et al., 2022), tumor necrosis factor *α* (TNF-α), high mobility group protein B1 (HMGB1) (Chu et al., 2017), or interleukin (IL)-1β (Shentu et al., 2021), which act in an autocrine or paracrine manner and contribute to peritoneal fibrosis by stimulating the proliferation of resident fibroblasts and ECM component deposition, and by inducing MMT of mesothelial cells, which further increases the number of peritoneal myofibroblasts. IL-6 is a vital inflammatory factor in the peritoneal cavity of patients undergoing PD. Stimulation of peritoneal mesothelial cells (PMCs) with the IL-6 and soluble IL-6 receptor complex (IL-6/S) has been shown to promote the EMT of PMCs in a STAT3-dependent manner (Yang et al., 2020). Additionally, the activation and proliferation of resident fibroblasts in the peritoneal membrane may be a reactive response to mesothelial cell damage.

During peritoneal membrane stress, changes in the metabolic status of mesothelial cells can exert effects on the early activation of fibroblasts. Si et al. (Si et al., 2019) evaluated the single-cell transcriptome of mesothelial cells obtained from the effluent of normal peritoneal biopsy and PD treated patients. The results demonstrated that mesothelial cells derived from the PD effluent are potentially a source of ECM components contributing to fibrosis because they demonstrate robust production of collagens (COL1A1 and COL4A1) and can undergo transformation into (myo)fibroblasts. Compared with normal mesothelial cells, mesothelial cells from patients in long-term PD highly expressed the key glycolysis enzymes, such as hexokinase 1 (HK1), HK2, and fructose-2, 6-biphosphatase 3, which is characterized by hyperglycolysis. Blockade of hyperglycolysis with 2-deoxyglucose suppressed profibrotic cellular phenotype and peritoneal fibrosis in mice. Another study (Su et al., 2023) which analyzed the above scRNA-seq data proposed that reduction of fatty acid oxidation in mesothelial cells characterizes patients on long-term PD. Cell trajectory analysis showed that COL1A1+ mesothelial cells expressed vimentin and FN1, supporting that mesothelial cells underwent transitions to fibroblasts. Restoration of fatty acid oxidation by overexpressing carnitine palmitoyl transferase 1A in PD mice reversed mesothelial cells transformation and reduced fibrotic lesions in the peritoneum.

In addition to the previously mentioned factors, other paracrine-signaling molecules include nucleic acids, specifically regulatory RNA or DNA. These nucleic acids can function as paracrine mediators between cells, influencing cellular communication and signaling processes. MicroRNA (miRNA) and long noncoding RNA (lncRNA), important regulators of gene expression, serve as cell-cell communicators in several kinds of peritoneal damage. These RNA molecules can impact the secretion of cytokines and growth factors by a cell, as well as elicit direct signaling responses in recipient cells following their secretion (Wang Z. K. et al., 2020; Guo et al., 2020). For instance, high-glucose (HG) treatment was shown to significantly upregulate the expression of the miR-199a-5p/214-3p gene cluster to induce downregulation of claudin-2 and E-cadherin related to EMT, whereas silencing of miR-199a-5p or miR-214-3p inhibited HG-induced mesothelial phenotypic transition, thereby reducing fibroblast formation (Guo et al., 2020). Similarly, miR-153-3p (Li D. et al., 2018), miR-129-5p (Xiao et al., 2015), miR-145 (Wu et al., 2019), and miR-200a (Wei et al., 2019a) target MMT to intervene in mesothelial-to-fibroblast transformation. The role of lncRNAs as paracrine-signaling mediators in the peritoneal membrane has attracted significant research attention. LncRNAs, despite not encoding proteins, play crucial roles in regulating gene expression and have been implicated in various biological processes. In PDF-induced peritoneal fibrosis mouse models, 127 lncRNAs are upregulated and 105 lncRNAs are downregulated (Liu Y. L. et al., 2015). LncRNA AV310809 could promote TGF-β1-induced MMT of PMCs via activation of the Wnt2/beta-catenin signaling pathway (Wei et al., 2019b). AK089579 inhibits the migration and MMT of PMCs in mice by suppressing the JAK2/STAT3 signaling pathway (Zeng et al., 2019). Most miRNAs and lncRNAs act on MMT to mediate mesothelial and fibroblast crosstalk.

Notably, extracellular vesicles (EVs) derived from tissues can modify their molecular cargo in response to changes in the environment, allowing them to mediate intercellular communication and play a crucial role in organ fibrosis. Huang et al. (2023a) performed four-dimensional label-free quantitative liquid chromatography-tandem mass spectrometry proteomic analyses on EVs from normal peritoneal tissues and PD-induced fibrotic peritoneum in mice for the first time. They found alterations in the concentration and protein composition of EVs between the normal control and PD groups. Proteins upregulated in PD EVs were predominantly involved in processes such as the response to injury and migration of leukocytes, which are associated with the development of fibrosis. EVs enriched with ILK, derived from injured mesothelial cells, can activate resident fibroblasts and promote peritoneal fibrosis (Huang et al., 2023b). Thus, EVs could potentially serve as a therapeutic target in the treatment of peritoneal fibrosis.

### 2.2 Pericyte-(Myo)fibroblast crosstalk

Pericytes are widely distributed on the basement membrane side of endothelial cells in the systemic microvascular system. The nucleus is oval in shape and the cytoplasm is stretched outward, extending along the long axis of microvessels to the outer lateral walls of multiple endothelial cells, enveloping the microvessels. Pericytes maintain the stability of blood vessels by interacting with endothelial cells and the basement membrane through paracrine signals (Attwell et al., 2016; Wimmer et al., 2019). Hepatic stellate cells (HSCs) are resident non-parenchymal liver pericytes that are the main actors in the pathogenesis of liver fibrosis (Trivedi et al., 2021). Single-cell transcriptomics analyses have uncovered the spatial zonation of HSCs across the hepatic lobule, revealing the ability of the HSCs to transdifferentiate into myofibroblasts during liver injury (Dobie et al., 2019). In the process of idiopathic pulmonary fibrosis, human lung pericytes transition into myofibroblast-like cells in the absence of TGF-β signaling (Yamaguchi et al., 2020). Although all myofibroblast precursor cells are not fully identified, it is clear that pericytes are an important source of myofibroblasts.

Activated pericytes dissociate from the microvasculature and transdifferentiate into myofibroblasts, which are the precursors of interstitial myofibroblasts (Yang et al., 2019). Pericyte separation causes structural deterioration and increased microvascular permeability, which may be a pathological sign of fibrosis (Wang M. et al., 2020). In the presence of PD, peritoneal microvascular pericyte loss and vascular structural and functional degradation take place. During angiogenesis, the exchange of cytokines occurs between pericytes and endothelial cells. PDGF-BB, PDGF-DD, endothelin-1 (ET-1), and TGF-β—produced by endothelial cells—act as the primary catalysts for pericyte activation, which results in pericyte damage and stimulates the transition of pericytes into a myofibroblast phenotype (Tang L. et al., 2022). As the marker of differentiated myofibroblasts, α-SMA is an important indicator of PMT. Primary pericytes that had been pretreated with TGF-β were found to have significantly higher levels of α-SMA expression, suggesting that the pretreated pericytes had undergone some degree of activation and changed into a myofibroblast phenotype.

### 2.3 Macrophage-(Myo)fibroblast crosstalk

There are many risk factors for inflammation in PD patients, such as uremic toxins, endotoxemia, and fluid overload (Li et al., 2017). Chronic inflammation is an important mechanism underlying the occurrence and maintenance of peritoneal fibrosis. This process occurs in relation to the overproduction of various proinflammatory factors (including TNF-α, IL-β1, IL-6, and monocyte chemoattractant protein-1 [MCP-1]) and infiltration of multiple inflammatory cells (including macrophages) (Wang et al., 2016).

Macrophages are one of the main immune cells, involved in the tissue damage and remodeling without bacterial inflammation. Activated macrophages regulate fibrogenesis by secreting cytokines and growth factors that modulate the proliferation and collagen synthesis of fibroblasts (Li Q. et al., 2018). Activated macrophages can produce a wide range of paracrine-signaling cytokines in the peritoneum, thus stimulating the proliferation of neighboring (myo)fibroblasts and synthesis of pathological extracellular matrix proteins such as collagen (Pai et al., 2020). Among these cytokines, various polypeptide growth factors, such as TGF-β1 and platelet-derived growth factor (PDGF), play an important role in the profibrogenic function of fibroblasts. However, activated macrophages also release metalloproteinases, which are enzymes responsible for degrading ECM components. This degradation process helps to counteract the excessive deposition of ECM and can exert a negative regulatory effect on fibrosis (Song et al., 2000). Transplantation of macrophages carrying the hepatocyte growth factor expression vector has been shown to significantly suppress submesothelial thickening and reduce type III collagen expression—that is, to prevent the progression of peritoneal fibrosis (Obata et al., 2023).

In recent years, the polarization of macrophages has attracted much attention. Macrophages can divide into two phenotypes—a proinflammatory phenotype (M1 or classical activation) or a pro-fibrotic phenotype (M2 or alternative activation)—depending on their distinct cytokine profiles and behavior after activation (Li Q. et al., 2018; Pai et al., 2020).

Macrophages displaying the M1 phenotype are key effector cells in pathogen clearance. Many studies have focused on the effects of M1 macrophages on fibroblasts. Early study (Song et al., 2000) suggests that the inhibitory effects of M1 macrophages on fibrogenic activities include reducing fibroblast proliferation and collagen biosynthesis, as well as enhancing collagen degradation. M1 macrophages induced by lipopolysaccharide (LPS) or interferon *γ* (IFN-γ) have been reported to express elevated levels of TNF-α and matrix metalloproteinase-7 (MMP-7) (Terri et al., 2021). MMP-7 can break down the extracellular matrix by digesting casein, gelatines, fibronectin and proteoglycan (Yin et al., 2021). MMP-7 has also been found to promote pulmonary and renal fibrosis through the facilitation of epithelial mesenchymal transition (Ke et al., 2017). Current research only indicates that MMP-7 is associated with the prognosis of peritoneal dialysis (Yin et al., 2021; Xiao et al., 2023), and its correlation with peritoneal fibrosis needs further investigation. In addition, it is worth noting that recent evidences point towards an active role of the M1 subtype in the genesis of peritoneal fibrosis (Terri et al., 2021). M1 macrophages and the cytokines they generate, which are present during the early stages of chronic inflammation, may contribute to parenchymal and matrix damage in the inflammatory tissue, potentially predisposing it to subsequent fibrosis. Research shows that peritoneal dialysis fluid (PDF) promotes the expression of Toll-like receptors (TLRs) in M1 phenotype macrophages, which seem to promote a proinflammatory milieu that exacerbates peritoneal injury and augments the decrease in peritoneal filtration function, thereby stimulating fibroblasts and promoting peritoneal fibrosis (Li Q. et al., 2018). The inhibition of the protein kinase C beta pathway aggravates peritoneal damage and fibrosis via M1 macrophages polarization in a murine model of PD (Balzer et al., 2019). *Lactobacillus casei Zhang*, a probiotic strain isolated from traditional fermented koumiss, has been shown to effectively reduce macrophage infiltration, inflammatory M1 polarization, and inflammatory cytokines in PD effluents to ameliorate peritoneal fibrosis in experimental mice (Wu et al., 2023).

M2 cells are critical to tissue remodeling, angiogenesis, and fibrogenesis, and are also involved in the fibrosis process. Bellon T et al. demonstrated that the ability of macrophages to stimulate fibroblast proliferation is related to the level of CC motif chemokine ligand 8 (CCL8) mRNA. Continuous stress due to PDF and infection may promote the infiltration of activated M2 phenotype cells and contribute to fibrotic processes through the secretion of TGF-β, CCL18, MMP-9, and other fibrogenic factors (Bellon et al., 2011). White J.C et al. reported an *in vitro* culture system to study macrophage-fibroblast interactions, in which media collected from macrophage cultures was used to culture normal human peritoneal fibroblasts (White et al., 2011). Chen YT et al. demonstrated that macrophages express CCL17, which stimulates migration and collagen production of fibroblasts in culture (Chen et al., 2020). Furthermore, histone deacetylase 8 (HDAC8) is a crucial enzyme for controlling the polarization of M2 macrophages via the STAT6 and PI3K/Akt signaling pathways. HDAC8 promotes EMT by inducing the phosphorylation of EGFR, which conversely, leads to the activation of its downstream fibrotic signaling pathways, including STAT3/HIF-1α and ERK1/2 (Zhou et al., 2023).

Fibroblasts can also act on macrophages in few researches. Buechler MB et al. shows that fibroblasts secrete various forms of retinoic acid to promote the expression of GATA binding protein 6 (GATA6) in macrophages. GATA6 is a transcription factor that macrophages rely on for their homeostasis, function, and localization (Buechler et al., 2019). Moreover, there is accumulating evidence confirming that macrophages can acquire fibroblast progenitor properties in the field of fibrotic disease research (Ito et al., 2022). Macrophages play a direct fibrotic role in fibrosing diseases including renal fibrosis (Tang P. M. K. et al., 2022) and lung fibrosis (Yang et al., 2021), via transition into myofibroblasts in a process named macrophage-to-myofibroblast transition. Cells undergoing macrophage-to-myofibroblast transition can be identified by the co-expression of macrophage (CD68) and myofibroblast (α-SMA) markers. In the interstitial fibrosis in chronic renal allograft injury, macrophage-to-myofibroblast transition cells account for approximately 50% of the myofibroblast population, and their quantity is associated with the severity of interstitial fibrosis. Knockout of Smad3 significantly reduce the number of these cells, and protected against interstitial fibrosis in renal allografts (Wang et al., 2017). In the study of lung fibrosis in rats with unilateral ureteral obstruction (UUO), compared with the sham group, the rats in the UUO group had large numbers of CD68+α-SMA + cells in severe interstitial fibrosis areas. Eplerenone, a mineralocorticoid receptor blocker, decreased UUO-induced macrophage-to-myofibroblast transition cells and attenuated lung inflammation and fibrosis (Yang et al., 2021). However, there is no direct evidence to suggest that macrophage-myofibroblast transition is involved in the progression of peritoneal fibrosis, which requires further experimental proof.

Taken together, differential macrophage activation is associated with opposing regulation of fibrogenesis. In turn, fibroblasts can regulate macrophage homeostasis and migration. Interactions between macrophages and fibroblasts jointly regulate peritoneal fibrosis.

### 2.4 Macrophage-mesothelial cell crosstalk

PMCs are epithelial-like cells, resting on a thin basement membrane that lines the entire surface of the abdominal cavity, including the internal organs (visceral peritoneum) and the body wall (parietal peritoneum) (Liu Y. et al., 2015). A monolayer of mesothelial cells is the first barrier against external injury factors (Zhao et al., 2020). Mesothelial cells play a crucial role in maintaining peritoneal homeostasis by preserving serosal integrity, promoting tissue repair, facilitating fibrin deposition and removal, engaging in antigen presentation, and synthesizing antifriction molecules. Under specific *in vitro* conditions, mesothelial cells have the ability to differentiate into myofibroblasts, smooth muscle cells, adipocytes, and osteoblasts. Upon exposure to injury or stimulation with fibroblast-associated growth factors, mesothelial cells can undergo a process called MMT (do Amaral et al., 2017). PD-related peritoneal fibrosis mainly involves PMCs, and the main mechanisms include abnormal expression of TGF-β1, MMT, and angiogenesis (Masola et al., 2022).

Macrophage cells are also located near the peritoneum (Helmke et al., 2019; Ivanov et al., 2019). In the early stage of peritoneal failure, large numbers of macrophages, monocytes, and other cells infiltrate the peritoneal tissue. Under the stimulation of various inflammatory factors such as IL-6 and TNF-α, PMCs are the first to be damaged; this is accompanied by the activation of local T and B cells and the recruitment of macrophages, which activates TLRs on the surface of PMCs. This triggers TLR/MyD88 signal transmission and activates downstream signals such as JNK, ERK, and NF-κB to induce the response to peritonitis. In this process, macrophage signaling is also altered, which further amplifies peritonitis, causes peritoneal injury and angiogenesis, and promotes MMT of PMCs. The researchers found that after coculture with M1-type macrophages, PMCs lost their typical epithelial cell morphology, which was accompanied by decreased E-cadherin and increased α-SMA production; in other words, PMCs underwent MMT. The underlying mechanism involved MMT of PMCs in direct contact with M1 macrophages, through activation of the Toll/IL-1 receptor domain containing adaptor via induction of the interferon-β (TRIF)-dependent TLR4 signaling pathway (Shi et al., 2016). Additionally, the activation of protein kinase C (PKC)-α in PMCs can cause upregulation of TGF-β and negative regulation of PKC-β. PKC-β deficiency causes proinflammatory M1 polarization in peritoneal macrophages, which in turn drives upregulation of PKC-α and TGF-β-mediated peritoneal fibrosis in PMCs (Balzer et al., 2019).

During inflammation-induced MMT, mesenteric mesothelial cells were observed to start expressing macrophage markers (OX43, ED1) and the number of macrophages appearing in the peritoneal cavity was found to increase dramatically. This observation raised the possibility that mesothelial cells might transdifferentiate into macrophage-like cells (Zsiros and Kiss, 2020). Subsequently, Katz S et al. showed that mesenteric mesothelial cells can differentiate into macrophages. This transdifferentiation may depend on special stimuli such as inflammation and granulocyte-macrophage colony-stimulating factor (GM-CSF) treatment. Under these stimuli, transdifferentiation of mesothelial cells into macrophages is regulated by the caveolin-1/ERK1/2/EGR1 signaling pathway (Katz et al., 2018; Zsiros and Kiss, 2020). In addition, interactions between mesothelial cells and macrophages contribute to peritoneal macrophage homeostasis. Ivanov S et al. suggests that culture of mesothelial cells with macrophages facilitates macrophage proliferation and survival. Mesothelial cells produce membrane-bound and secreted CSF1, both of which sustain peritoneal macrophage growth (Ivanov et al., 2019).

Several additional studies propose other mechanisms for the interaction between macrophages and mesothelial cells. Helmke et al. (2019) shows that the macrophage-mesothelial cell interaction upregulates mesothelial TGF-β and CX3CL1 expression in a CX3CR1-dependent manner in a PD mouse model. The expression of the chemokine receptor CX3CR1 by peritoneal macrophages can enhance the role of its ligand CX3CL1 in PMCs, and induce PMCs to produce TGF-β. TGF-β in PMCs further upregulates the expression of CXCR1 in macrophages, inducing macrophage-mesothelial intercellular crosstalk to promote chronic inflammation and peritoneal fibrosis.

In conclusion, MCs line the entire surface of the abdominal cavity and macrophages are located nearby. There is interaction between the two types of cells during peritoneal fibrosis associated with PD. M1 macrophages can regulate the transformation of mesothelial cells into mesenchymal cells. Likewise, mesothelial cells can transdifferentiate into macrophage-like cells and sustain macrophage growth.

### 2.5 Endothelial cell-mesothelial cell crosstalk

In vessels, endothelial cells typically form a cobblestone-like monolayer of inactive cells that lines the luminal surface of vascular tubes and controls vessel barrier function, and thereby influences peritoneal matrix transport and ultrafiltration (Herbert and Stainier, 2011; Herzog et al., 2020). Crosstalk between endothelial cells and other cells of the peritoneum is based on peritoneal capillaries—the main site for the exchange of materials between blood components and peritoneal dialysate components—to remove metabolites and correct water-electrolyte and acid-base balance disorders. Even though the endothelial cells are never in direct contact with the PDF, the submesothelial vasculature is significantly more damaged than expected by uremia alone (Sacnun et al., 2022). Endothelial cells are the main target cells of VEGF. VEGF-mediated angiogenesis is the major pathological mechanism for the loss of ultrafiltration capacity in PD patients. Long-term non-physiological PDF stimulation, uremic state, peritonitis, and other micro-inflammatory states lead to vascular morphological changes in the peritoneum, which are characterized by higher vessel density, vascular wall thickening, and vasodilation of capillaries (Qayyum et al., 2015; Shi et al., 2022).

VEGF, which is produced by peritoneal cells stimulated by PD-related factors, is an endothelial-specific growth factor and plays a dominant role in mediating endothelial cell sprouting, proliferation, migration, and vascular permeability. Zhu X et al. reported that VEGF/VEGFR mediates mesothelial-endothelial crosstalk and promotes peritoneal angiogenesis during PD (Zhu et al., 2021). Mesothelial cells that have undergone MMT are the main source of VEGF in PD patients (Aroeira et al., 2005). The materials used for PD (i.e., peritoneal dialysate, dialysis catheter, pipe, and package) are all non-biocompatible substances. High glucose and excessive advanced glycation end products (AGEs) can promote VEGF release from PMCs.

Combined incubation of PMCs with the anti-RAGE (AGE receptor) antibody downregulates capillary tube formation of human umbilical vein endothelial cells (HUVECs), which explains the vasoformation in PD patients with VEGF production by PMCs through mesothelial RAGE activation. Similarly, glucose degradation products also increase the expression of VEGF in cultured PMCs (Shi et al., 2022). In a coculture system of PMCs and HUVECs, IL-6/S was shown to mediate the production of VEGF and angiopoietins to downregulate the expression of endothelial junction molecules, thereby affecting vascular permeability (Yang et al., 2020).

In addition to the VEGF signaling pathway, a few other pathways have been found to mediate mesothelial-endothelial crosstalk. Zhu N et al. reported that the increase of ET‐1, produced predominantly by endothelial cells, could promote HPMC proliferation via the nuclear transcription factor Ets‐1. ET-1 is also involved in VEGF production, which in turn acts on endothelial cells (Zhu et al., 2019).

### 2.6 Endothelial cell-(myo)fibroblast crosstalk

Endothelial-mesenchymal transition (EndoMT)EndoMT is one of the important pathogeneses of peritoneal fibrosis, from which approximately 3%–5% of submesothelial fibroblasts in peritoneal dialysis originate (González-Mateo et al., 2015). Only a few studies on peritoneal fibrosis focus on endothelial cell-(myo)fibroblast crosstalk, proposing. EndoMT is an intricate biological process during which endothelial cells undergo a transition, losing their specific markers and adopting a mesenchymal or (myo)fibroblastic phenotype. This transition involves the expression of mesenchymal cell products such as α-SMA, COL1, and fibroblast-specific protein (FSP)-1 (Piera-Velazquez et al., 2011).

Peritoneal hyalinizing vasculopathy (PHV) is pathologic manifestation of long-term peritoneal dialysis, especially in patients with capsular peritoneal sclerosis. Peritoneal biopsies from PHV samples showed co-expression of endothelial and mesenchymal markers, indicating an ongoing process of EndoMT (Díaz et al., 2020). Exposure of the peritoneum to peritoneal dialysis fluid induces endothelial phenotype changes into myofibroblast like cells through Smad3 activation, leading to abnormal accumulation of collagen IV in the peritoneal vascular wall. These processes may lead to vascular occlusion, and subsequent membrane dysfunction and ultrafiltration failure. Rapamycin can inhibit angiogenesis by reducing the production of VEGF or blocking its receptor. In PD rat model, rapamycin can reduce submesothelial CD31 vessels and the number of CD31+FSP1+endothelial cells, indicating that rapamycin decreased PD-induced angiogenesis and Endo-MT (González-Mateo et al., 2015).

However, there are few studies on the mechanism of endothelial mesenchymal transition in PD-related peritoneal fibrosis. In fibrotic diseases, the major regulator of EndoMT is TGF-β signaling. In the study of diabetic cardiomyopathy, silencing miR-195-5p targets inhibits TGF-β1-Smads pathway by targeting Smad7, thus inhibiting EndoMT and alleviating myocardial fibrosis (Ding et al., 2021). In the study of pulmonary fibrosis, PM_2.5_ induces EndMT by regulating the TGF-β1/Smad3 pathway (Ma et al., 2020). TGF-β1 levels were increased in both peritoneal tissue and effluent of PD mice (Su et al., 2023). It is possible that the TGF-β signaling pathway is involved in endothelial-mesenchymal transition in peritoneal fibrosis.

### 2.7 Endothelial cell-adipocyte crosstalk

Peritoneal adipose tissue is abundant in the omental or mesenteric peritoneum. Loose adipose tissue with peritoneal capillaries forms the deepest layer of the peritoneal membrane (Lai and Leung, 2010; Shi et al., 2017). Interactions between endothelial cells and adipocytes are mostly mediated via paracrine factors and direct cell-cell contacts by abundant microvessels contained in adipose tissue.

Endothelial cells play crucial roles not only in the blood supply, but also in the production of, endothelium-derived relaxing factor and cytokines. Endothelial cells secrete numerous cytokines, including angiogenic factors (VEGF (Carter et al., 2015)), inflammatory factors (IL-6 (Gopinathan et al., 2015)), chemokines (MCP-1 (Cakmak et al., 2012)), and adhesion molecules (intercellular cell adhesion molecule-1, ICAM-1 (Mosbah et al., 2016)). These factors can participate in inflammation, extracellular matrix formation, and angiogenesis, through various mechanisms.

Peritoneal adipose tissue is deeply embedded in the mesothelial monolayer and subendothelial layer, which is formed by mature adipocytes and stromal vessels. Adipocytes can mediate various physiological processes through secretion of an array of mediators and adipokines, including leptin, TNF-α, IL-6, TGF-β, VEGF, and other growth factors (Lai and Leung, 2010). Moreover, adipocytes express receptors for leptin, IGF-1, TNF-α, IL-6, and TGF-β, and may form a network of local autocrine, paracrine, and endocrine signals. Excessive long-term sugar intake due to glucose-containing peritoneal dialysis fluid leads to fat accumulation in PD patients, and the accumulated fat is often found in the mesentery or omentum (Aoki et al., 2013). Klotho is a molecule involved in lipid metabolism. *In vivo* experiments have also revealed that GSK343 (enhancer of zeste 2 polycomb repressive complex 2 subunit (EZH2) inhibitor) could relieve peritoneal fibrosis, lipid deposition, and EMT by mitigating EZH2 and restoring klotho expression (Wang et al., 2023). During PD, following damage to the mesothelial monolayer, dialysate can also damage adipose tissue.

During the process of PD, patients undergo chronic low-grade inflammation in dialysate and plasma, which stimulates both adipose tissue and angiogenesis/neoangiogenesis involving endothelial cells. Cytokines released by adipocytes can act on endothelial cells, which are the main mediators of their crosstalk. TNF-α has been shown to mediate endothelial dysfunction (Catar et al., 2013). IL-6 can promote VEGF directly (Avraham-Davidi et al., 2013). Leptin, the most abundant hormone secreted by adipocytes, can indirectly promote angiogenesis (Garonna et al., 2011). All the processes mentioned above can lead to peritoneal fibrosis and eventually peritoneal ultrafiltration failure.

Likewise, in PD-related peritoneal neoangiogenesis, macrophages interact with endothelial cells through the secretion of VEGF from macrophages present in the peritoneal fluid. The utilization of conditioned medium derived from macrophages isolated from the ascites of patients with spontaneous bacterial peritonitis has been found to significantly augment VEGF expression and facilitate heightened proliferation of endothelial cells. The proangiogenic properties of M2-polarized macrophages rely on their ability to produce angiogenic cytokines, including VEGF, TGF-β, and matrix metalloprotein-9. M2 macrophages are the primary subtype involved in peritoneal injury, highlighting their contribution to peritoneal neoangiogenesis and fibrosis (Shi et al., 2022).

### 2.8 Mast cells

Mast cells are tissue-resident, innate immune cells that play a crucial role in both the inflammatory response and tissue homeostasis. These cells contain specialized cytoplasmic granules that store a range of inflammatory mediators, including histamine, proteases, cytokines, and chemotactic factors (Anaf et al., 2006). Upon activation, mast cells release and secrete these inflammatory molecules to increase vascular permeability, trigger the contraction of smooth muscles, and attract and activate numerous inflammatory cells (Malbec et al., 2007). In tumor-related studies, mast cells have been shown to exert regulatory influence over the tumor microenvironment by modulating several processes, including cell proliferation and survival, angiogenesis, invasiveness, and metastasis (Aponte-Lopez and Munoz-Cruz, 2020). Mast cells, which may facilitate organ fibrosis, are known to accumulate in significant numbers within the peritoneum. Thus, mast cells likely contribute to the pathogenesis of peritoneal fibrosis (Kazama et al., 2015).

In normal peritoneal tissue, mast cells are localized submesothelially and barely visible by hematoxylin-eosin staining. Once subjected to peritoneal dialysis-related stimuli, mast cells become activated and cause peritonitis and fibrosis associated with PD. Alscher et al. (2007) found that mast cell numbers increase in inflammatory diseases of the peritoneum and in PD patients. Mast cell degranulation is observed in all altered peritoneal tissues compared with the unstressed normal peritoneum. Besides, these authors demonstrated a negative and highly significant correlation between mast cell number and fibrosis, and a positive correlation between mast cell number and number of vessels in the peritoneum.

In the fibrotic peritoneum of chronic renal failure rats, a notable rise was observed in the mast cell population within both the parietal and visceral layers of the peritoneum. These mast cells were found to exhibit heightened production of fibroblast-activating factors, which stimulate collagen synthesis. It is believed that the proliferation and increased activity of mast cells in the peritoneum play a crucial role in driving the progression of peritoneal fibrosis (Kazama et al., 2015). In summary, mast cells interact with vascular endothelial cells and fibroblasts to increase vascular permeability, stimulate collagen synthesis, and promote the fibrosis process.

### 2.9 Other immune cells

The immune response is divided into the innate and adaptive responses. Innate immunity encompasses neutrophils, monocytes, macrophages, complement, cytokines, and acute phase proteins, that provide immediate host defense. Adaptive immunity consists of antigen-specific reactions through T lymphocytes (T-cells) and B lymphocytes (B cells) (Parkin and Cohen, 2001). The peritoneum has three distinctive layers: the mesothelium, a basal lamina, and the submesothelial stroma. The submesothelial stroma is an important source of immune cells (van Baal et al., 2017). During PD, the onset of peritonitis is one of the most serious complications. Peritoneal fibrosis is the end point of a progressive alteration of the peritoneal membrane due to a wide array of inflammatory and infectious events. Immune cells, crucial participants in this process, have a significant impact on the development of peritoneal fibrosis (Zhou et al., 2016; Terri et al., 2021).

The differentiation of T-cells plays a pivotal role in immune and inflammatory responses, and the modulation of T-cell activity may serve as a therapeutic approach to mitigate peritoneal damage. T-cells can be broadly categorized into two distinct functional groups: CD4^+^ T helper cells (Th) and CD8^+^ cytotoxic T lymphocytes (Wik and Skalhegg, 2022). Among T helper subsets, in addition to the classical Th1 and Th2 cells, regulatory T (Treg) and T helper 17 (Th17) cells have gained attention. Th17 lymphocytes have been identified as key contributors to peritoneal fibrosis (Marchant et al., 2020). Th17 cells predominantly release cytokines of the IL-17 family, particularly IL-17A. Multiple lines of experimental evidence underscore the involvement of Th17/IL17A in both acute peritoneal damage and chronic inflammation of the peritoneal membrane (Trionfetti et al., 2023). Spontaneous and cytokine-mediated Th17 polarization is enhanced by PD-range glucose concentrations. Exposure to PDF results in the localized production of Th17-related cytokines, including IL-17A and IL-6. Notably, a correlation has been observed between peritoneal IL-17A protein levels and peritoneal membrane thickness. Antibody blockade of the IL-17A pathway significantly reduces experimental peritoneal fibrosis (Helmke et al., 2021). The imbalance between the populations of Th17 and Treg cells is one of the factors involved in the pathogenesis of peritoneal fibrosis (Lin et al., 2019). The main cytokines involved in Th17/Treg balance are TGF-β and IL-6. TGF-β in the absence of IL-6 induces Foxp3, thus directing T-cell differentiation away from the Th17 transcriptional program and decidedly toward the Treg lineage (Liappas et al., 2015). In the context of peritoneal injury, animal models exposed to dialysis fluids have demonstrated an imbalance in the Th17/Treg cell ratio, with a predominance of Th17 cells, which has been associated with peritoneal damage during PD (Marchant et al., 2020).

Fat-associated lymphoid clusters (FALCs) are a unique anatomical structure of the peritoneum, specifically localized in the omentum. FALCs are identified leukocyte aggregates, mainly composed of B and T lymphocytes and stromal cells, designed to maintain peritoneal homeostasis and mitigate local inflammation (Han et al., 2022). These clusters of leukocytes possess the unique capacity to collect fluids, particles, and cells from the peritoneal cavity (Buscher et al., 2016; Cruz-Migoni and Caamano, 2016; Liu M. et al., 2021; Terri et al., 2021). Peritonitis induces FALCs formation that is dependent on TNF production by monocytes and/or macrophages and TNF receptor signaling in stromal cells (Jackson-Jones et al., 2020). The chemokine CXCL13, which is produced by mesothelial cells, controls the migration of B1 cells to FALCs. CCL19, another chemokine, contributes to the recruitment of monocytes during inflammatory processes. Interactions between CCL19-producing FALCs and inflammatory monocytes facilitate T-cell-dependent B-cell immune responses. As such, FALCs play a key role not just in inflammation but also in the generation of the adaptive immune response that follows (Cruz-Migoni and Caamano, 2016; Jackson-Jones et al., 2020; Terri et al., 2021).

## 3 Inter-organ communication

### 3.1 Crosstalk between peritoneal membrane and gut

The peritoneal membrane starts developing alongside the primitive gut in early embryonic development, which leads to formation of the structure of the abdominal cavity. Also, the peritoneal membrane, especially the mesentery, and gut are close to each other in anatomical positions (Bermo et al., 2020); consequently, they can interact in both physiological and pathological conditions. In peritoneal fibrosis, gut barrier dysfunction can affect the dialysis efficiency and the stability of the PD catheter. Gut diseases can lead to pathological changes of the peritoneal membrane such as intestinal tumors, intestinal obstruction, and Crohn’s disease. However, under common conditions, PD is not recommended for end-stage renal disease patients with intestinal diseases; therefore, this is not explored further here.

In PD patients, the inflammatory state of microinflammation and peritonitis leads to numerous pathological changes, including increased gut mucosa permeability, gut barrier function changes, and translocation of gut bacteria. The imbalance of microbiota increases the translocation of endotoxins and uremic toxins by altering the gut barrier, leading to inflammation and oxidative stress, and inducing profibrotic effects (Shimizu et al., 2013; Tang et al., 2015; Ramezani et al., 2016).

Elevated levels of uremic toxins derived from the gut microbiome, such as indoxyl sulfate, p-cresol sulfate, and trimethylamine N-oxide, have been observed in patients with impaired kidney function. These uremic toxins are bound to proteins and are not efficiently eliminated through dialysis, which has led to suggestions of their association with chronic inflammation. Probiotics have shown promise in reducing the serum concentrations of these uremic toxins and improving the inflammatory status in patients undergoing dialysis (Jiang et al., 2021). Furthermore, long-term PD results in low appetite, malnutrition, atrophy of gut mucosa epithelial cells, and decreased peristalsis function, which leads to microbial shift, endogenous peritoneal infection, and eventually peritoneal fibrosis.

Gut microbiota make up the largest microecosystem in the human body, and are of vital importance for maintaining homeostasis and immune function (Michaudel and Sokol, 2020). The diversity and community of gut microbiota differ remarkably between patients with chronic kidney diseases and those undergoing hemodialysis or PD (Michaudel and Sokol, 2020). Recent research indicates that the composition of the gut microbiota in PD patients is not solely determined by the PD treatment method itself. Instead, it is influenced by the individualized dialysis prescription and the clinical characteristics of each PD patient. Additionally, studies have established correlations between the gut microbiota composition and factors such as the duration of dialysis treatment (dialysis vintage), the level of residual kidney function, and the extent of peritoneal glucose exposure in PD patients (Stepanova, 2023). Prolonged dialysis sessions, elevated peritoneal glucose exposure, and the decline in residual renal function have been linked to changes in gut microbial composition and a decrease in the production of branched-chain short-chain fatty acids among PD patients. These results underscore the significance of minimizing the use of HG solutions and preserving residual renal function to uphold the equilibrium of gut microbiota, particularly in individuals undergoing extended periods of PD treatment (Jiang et al., 2021).

Loss of bacterial biodiversity is the most frequent outcome following gut dysfunction. Microbial diversity significantly decreases in PD patients, and the abundance of Firmicutes, Actinobacteria, and Proteobacteria is altered. Enterobacteriaceae and *Pseudomonas* are also increased in fecal samples of PD patients, which accounts for a bacteriological cause of peritonitis or severe peritonitis (Ren et al., 2020). Recent studies state that translocation of gut bacteria, such as anaerobic Gram-positive cocci (*Enterococcus*) (Adapa et al., 2019) and facultative anaerobe Gram-negative bacilli (*Citrobacter freundii*) (Adapa et al., 2020), directly or indirectly results in peritonitis in PD. Trimethylamine-N-oxide (TMAO), a metabolite produced by the gut microbiota, might be a risk factor for peritonitis in PD patients (Zhang et al., 2022). Zhang et al. (2022) found that serum TMAO levels were positively correlated with markers of systemic inflammation as well as peritoneal inflammation in PD patients. In chronic kidney disease rat models, TMAO was observed to significantly enhance inflammatory cell infiltration and the production of inflammatory cytokines within the peritoneum, induced by peritoneal dialysate.

## 4 Conclusion

In conclusion, the peritoneum is a complex structure with a variety of functions. During PD, multicellular interactions maintain peritoneal function and stability. Peritoneal cells, such as mesothelial cells, endothelial cells, macrophages, fibroblasts, pericytes, and mast cells, secrete cytokines, express proteins, and transdifferentiate into other cell phenotypes in response to the pathological factors underlying peritoneal fibrosis. The abovementioned cells and processes may serve as potential targets and represent new directions for intervention in intercellular communication and regulation of various cells in the treatment of peritoneal fibrosis.
